# Hospital Occupancy and Emergency Department Boarding During the COVID-19 Pandemic

**DOI:** 10.1001/jamanetworkopen.2022.33964

**Published:** 2022-09-30

**Authors:** Alexander T. Janke, Edward R. Melnick, Arjun K. Venkatesh

**Affiliations:** 1 Department of Emergency Medicine, Yale University School of Medicine, New Haven, Connecticut; 2 VA Ann Arbor, University of Michigan, National Clinician Scholars Program, Ann Arbor; 3 Center for Outcomes Research and Evaluation, Yale University, New Haven, Connecticut

## Abstract

This cross-sectional study uses national benchmarking data to evaluate hospital occupancy and emergency department boarding during the COVID-19 pandemic.

## Introduction

Emergency department (ED) boarding refers to holding admitted patients in the ED, often in hallways, while awaiting an inpatient bed. The Joint Commission identified boarding as a patient safety risk that should not exceed 4 hours.^1^ Downstream harms include increased medical errors, compromises to patient privacy, and increased mortality.^2^ Boarding is a key indicator of overwhelmed resources and may be more likely to occur when hospital occupancy exceeds 85% to 90%.^3^

Hospital resource constraints have become more salient during the COVID-19 pandemic and have been associated with excess mortality.^4^ Existing federal data fail to capture a comprehensive view of resource limitations inclusive of ED strain.^5^ We used a national benchmarking database to examine hospital occupancy and ED boarding during the COVID-19 pandemic.

## Methods

This cross-sectional study used aggregated hospital measures available through a voluntary peer benchmarking service offered by Epic Systems Corporation, an electronic health record vendor. Measures were collected monthly from January 2020 to December 2021. Annual ED visit volumes and total hospital beds for participating sites were included (eTable in the Supplement). We reported median and 5th to 95th percentile for hospital occupancy (percentage of staffed inpatient beds occupied, calculated hourly and averaged over the month), ED boarding time (median time from admission order to ED departure to an inpatient bed), and ED visit count. The study was classified as exempt by the institutional review board at Yale University because the study did not use patient data. This study followed the STROBE reporting guideline.

Distribution of ED boarding time was examined across hospital occupancy levels, with a threshold of 85% or greater based on Kelen et al.^3^ We plotted all 3 measures with new national daily COVID-19 cases.^6^ The difference in median ED boarding time between high-occupancy and low-occupancy hospital-months was evaluated using the Wilcoxon rank sum test .Analyses were performed using R, version 4.0.2.

## Results

**Figure 1.**
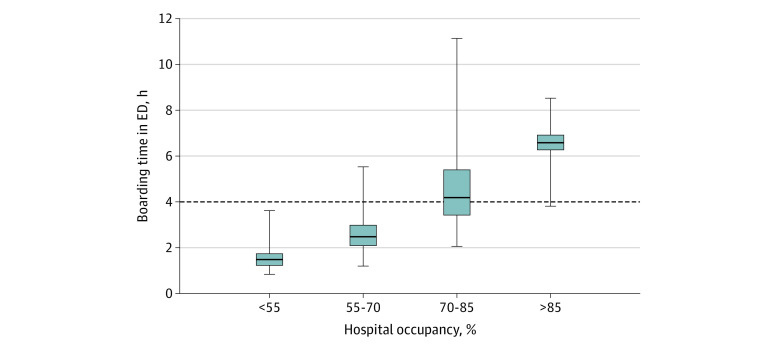
Median Boarding Time by Occupancy Across Sample Hospitals Data are from Epic Systems Corporation peer benchmarking. Center horizontal lines represent medians; lower and upper bounds of the boxes, 25th and 75th percentiles; vertical lines, 5th to 95th percentile; and dashed horizontal line, 4-hour standard set by The Joint Commission.

**Figure 2.**
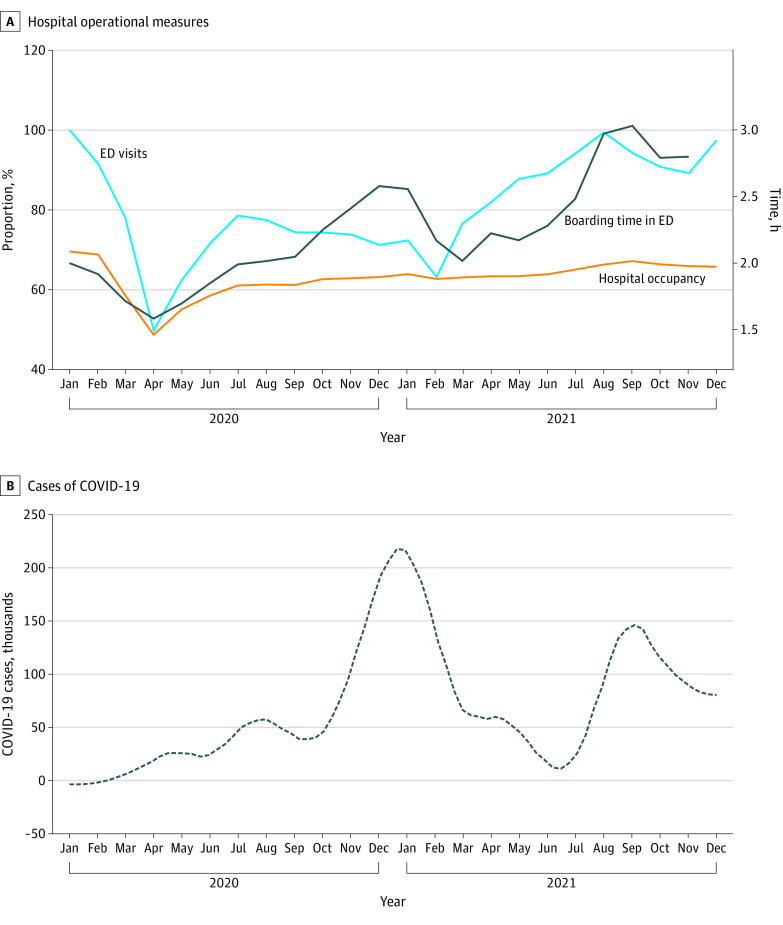
Hospital Operational Measures from January 2020 to December 2021 Data are from Epic Systems Corporation peer benchmarking. A, Hospital occupancy is the percentage of staffed beds; ED visits are from January 2020.

Hospitals reporting benchmarking data increased from 1289 in January 2020 to 1769 in December 2021. Occupancy rates and boarding time had a threshold association: when occupancy exceeded 85%, boarding exceeded The Joint Commission 4-hour standard for 88.9% of hospital-months (Figure 1). In those hospital-months, median ED boarding time was 6.58 hours compared with 2.42 hours in other hospital-months (*P* < .001). Across all hospitals, the median ED boarding time was 2.00 hours (5th-95th percentile, 0.93-7.88 hours) in January 2020, 1.58 hours (5th-95th percentile, 0.90-3.51 hours) in April 2020, and 3.42 hours in December 2021 (5th-95th percentile, 1.27-9.14 hours). Median hospital occupancy was highest in January 2020 (69.6%; 5th-95th percentile, 44.3%-69.6%), 48.7% (5th-95th percentile, 28.7%-69.9% hours) in April 2020, and 65.8% (5th-95th percentile, 42.7%-84.8%) in December 2021 (Figure 2).

## Discussion

We found that hospital occupancy greater than 85% was associated with increased ED boarding beyond the 4-hour standard. Throughout 2020 and 2021, ED boarding increased even when hospital occupancy did not increase above January 2020 levels. The harms associated with ED boarding and crowding, long-standing before the pandemic, may have been further entrenched. Study limitations were the inability to differentiate occupancy for specific services, median measures of boarding likely underestimated actual burden, and the sample was anchored to specific data fields within the Epic peer benchmarking service. Future research should explore more complex measures like staffing variability and local outbreak burden. Policy makers should address acute care system strain in future pandemic waves and other disasters to avoid further hospital system capacity strain and unsafe patient care conditions.

## Notes

Supplement. **eTable.** Sample Site Characteristics from the Epic Peer Benchmarking Service
