# Supplementary material for: Hospital Occupancy and Emergency Department Boarding During the COVID-19 Pandemic
Source: JAMA Netw Open. 2022 Sep 30;5(9):e2233964. doi: 10.1001/jamanetworkopen.2022.33964 (PMC9526134; doi:10.1001/jamanetworkopen.2022.33964)
Supplement: Supplement. — eTable. Sample Site Characteristics from the Epic Peer Benchmarking Service [file jamanetwopen-e2233964-s001.pdf]

Supplemental Online Content

Janke AT, Melnick ER, Venkatesh AK. Hospital occupancy and emergency department boarding during the COVID-19 pandemic. *JAMA Netw Open*. 2022;5(9):e2233964. doi:10.1001/jamanetworkopen.2022.33964

eTable. Sample Site Characteristics from the Epic Peer Benchmarking Service

This supplemental material has been provided by the authors to give readers additional information about their work.

© 2022 Janke AT et al. *JAMA Network Open.*

eTable. Sample Site Characteristics from the Epic Peer Benchmarking Service

**Hospital Beds**	Proportion	**Annual ED Visits**	Proportion
**<100**	42%	**0-20,000**	29%
**100-299**	28%	**20-40,000**	30%
**300-499**	14%	**40-60,000**	21%
**500-999**	12%	**60-80,000**	10%
**1000+**	4%	**80,000+**	10%

© 2022 Janke AT et al. *JAMA Network Open.*
